# Supplementary material for: Development and validation of a risk prediction model for hypoproteinemia after adult cardiac valve surgery: implications for clinical care
Source: PeerJ. 2025 Jul 16;13:e19676. doi: 10.7717/peerj.19676 (PMC12275900; doi:10.7717/peerj.19676)
Supplement: Supplemental Information 3 [file peerj-13-19676-s003.docx]

STROBE statement: list of necessary items for observational research

| **Author list:** Fang Wang, Zhenzhen Su, Xiaoqian Guo, Man Li, Rui Wang, Yanjun Xu | | | |
| --- | --- | --- | --- |
| First author's name: Fang Wang | | | Correspondence author: Fang Wang |
| Title of manuscript: Development and validation of a hypoproteinemia risk prediction model post-adult cardiac valve surgery: Implication for Clinical Care | | | |
| **Content and theme** | **Item number** | **Cross-check items (please tick if necessary)** | |
| **Title and abstract** | 1 | ☑ The research design should be expressed by common technical terms in the title or abstract.  ☑ The abstract should be rich in content, and can accurately and smoothly express what was done and found in the research. | |
| **Introduction** | | | |
| Background or principle | 2 | ☑ Explain the reported research background and principle. | |
| Target | 3 | ☑ Clarify the research objectives, including any predetermined assumptions. | |
| **Methods** | | | |
| Research design | 4 | ☑ State the elements of research design earlier in the paper. | |
| Research site | 5 | ☑ Describe the research site, specific place and relevant time range (including the time of collection, exposure, follow-up and data collection). | |
| Research objects | 6 | ☑ Cohort study: describe the eligibility criteria, source population and selection method of the selected subjects, and describe the follow-up method;  □ Case-control study: describe the eligibility criteria, source population and selection method for selecting confirmed cases and controls, and describe the principle of selecting cases and controls;  □ Cross-sectional study: describe the eligibility criteria, source population and selection method for selecting research objects.  □ Cohort study-paired study: describe the matching criteria and the number of exposed and unexposed;  □ Case-control study-paired study: describe the matching criteria and the number of controls corresponding to each case. | |
| Research variable | 7 | ☑ clearly define the outcome, exposure, predictors, potential confounders and effect modifiers (e.g. If possible, give the diagnostic criteria) | |
| Data source or measurement | 8* | ☑ For each concerned variable, describe its data source and detailed judgment (measurement) method (if There are multiple groups, and the comparability of judgment methods between groups should also be described) | |
| Bias | 9 | ☑ Describe and explain the process of potential bias. | |
| Sample size | 10 | ☑ Explain how to determine the sample size. | |
| Metric variable | 11 | ☑ Explain how to deal with the measurement variables in the analysis (if possible, describe how to select the grouping and the reasons for grouping) | |
| Statistical method | 12 | ☑ Describe all statistical methods, including methods for controlling confounding;  ☑ Describe subgroup and interaction inspection methods;  ☑ Describe the treatment method of missing values; | |
|  |  | ☑ cohort study: if possible, explain the handling method of lost visit;  Case-control study: if possible, explain the matching method of cases and controls; Cross-sectional study: if possible, describe the statistical methods determined according to the sampling strategy.  ☑ Describe sensitivity analysis | |
| **Results** | | | |
| Research objects | 13* | ☑ Report the number of subjects in each stage of the study, such as the number that may be qualified, the number that is tested to be qualified, the number that is confirmed to be qualified, the number that is included in the study, the number that has been followed up and the number that has been analyzed;  ☑ Describe the reasons why the subjects failed to participate in each stage;  ☑ Consider using a flowchart. | |
| Descriptive information | 14* | ☑ Describe the characteristics of the research object (such as demographic, clinical and social characteristics) and information about exposure and potential confounding factors;  ☑ Point out the number of subjects with missing values for each concerned variable;  □ Cohort study: summarize the follow-up time (such as average time and total time) | |

| Outcome data | 15* | ☑ Cohort study: report the number of final events or summarize the number of final events according to time;  □ Case-control study: report the quantity of each exposure category or the comprehensive indicators of exposure;  □ Cross-sectional study: report the number of outcome events or summarize the exposed measurement results. |
| --- | --- | --- |
| Main results | 16 | ☑ Give the estimated value and accuracy (such as 95%CI) of the correlation strength between uncorrected and corrected confounding factors, and clarify which confounding factors are adjusted according to and the reasons for choosing these factors;  ☑ Report the grouping boundary value when grouping continuous variables;  ☑ If there is any correlation, the relative risk in a meaningful period can be converted into absolute risk. |
| Other analysis | 17 | ☑ Report other analysis, such as subgroup and transactional analysis and sensitivity analysis. |
| **Discussion** | | |
| Important results | 18 | ☑ Summarize the important results related to the research hypothesis. |
| Limitations | 19 | ☑ Discuss the limitations of the research by combining the sources of potential bias and inaccuracy; Discuss the potential bias  Direction and size |
| Explain | 20 | ☑ Combining the research purpose, limitations, multi-factor analysis, similar research results and other relevant evidence,  Carefully give an overall explanation of the results. |
| Generalizability | 21 | ☑ Discuss the generalization of the research results (extrapolation validity) |
| **Other information** | | |
| Aid financially | 22 | ☑ Give the funding sources and sponsors of the current research (if possible, give the funding situation of the original research condition) |

* In case-control study, the information of case and control are given respectively; If possible, the information of exposed group and unexposed group is given in cohort study and cross-sectional study.
